# Efficacy and safety of aerosol inhalation of recombinant human interferon-α2b injection in children hospitalized with human adenovirus pneumonia: a prospective, multicenter, randomized, controlled trial in China

**DOI:** 10.1097/JS9.0000000000004635

**Published:** 2026-01-19

**Authors:** Hai-Feng Liu, Xue-Zu Zhang, Pei-Yan Li, Yong-Liang Luo, Xing-Ping Tao, Yang Xiao, Ming-Ze Sui, Jing Zhao, Chun-Yan Zhang, Ruo-Yu Niu, Ming-Juan Lin, Hong-Yan Zhu, Tao Yang, Mei Xiang, Hong-Min Fu

**Affiliations:** a Department of Pulmonary and Critical Care Medicine, Yunnan Medical Center for Pediatric Diseases, Kunming Children’s Hospital, Children’s Hospital Affiliated to Kunming Medical University, Kunming, China; b College of Pediatrics, Kunming Medical University, Kunming, China; c Department of Pediatrics, The People’s Hospital of Lincang, Lincang, China; d Department of Pediatrics, The People’s Hospital of Xishuangbanna Dai Autonomous Prefecture, Jinghong, China; e Department of Pediatrics, Maternal and Child Healthcare Hospital of Yunnan Province, Kunming, China; f Department of Respiration, Kaiyuan Children’s Hospital, Kaiyuan, China; g Yunnan Key Laboratory of Children’s Major Disease Research, Department of Otorhinolaryngology Head and Neck Surgery, Kunming Children’s Hospital, Children’s Hospital Affiliated to Kunming Medical University, Kunming, China; h State Key Laboratory of Phytochemistry and Plant Resources in West China, Kunming Institute of Botany, Chinese Academy of Sciences, Kunming, China; i Department of Pediatrics, The First People’s Hospital of Zhaotong, Zhaotong Hospital Affiliated to Kunming Medical University, Zhaotong, China; j Department of Respiration, Maternal and Child Healthcare Hospital of Qujing, Qujing, China; k Department of Pediatrics, Yan’an Hospital of Kunming, Yan’an Hospital Affiliated to Kunming Medical University, Kunming, China; l Department of Pediatrics, The First People’s Hospital of Honghe, Mengzi, China

**Keywords:** aerosol inhalation, children, human adenovirus, interferon-α2b, pneumonia, randomized controlled trial

## Abstract

**Background:**

Human adenovirus (HAdV) pneumonia represents one of the gravest threats to child health worldwide. However, effectively and safely antiviral strategies for HAdV infections remain limited. In this study, we aimed to comprehensively investigate the efficacy and safety of aerosol inhalation of recombinant human interferon-α2b (IFN-α2b) injection in children hospitalized with HAdV pneumonia.

**Methods:**

This randomized controlled trial recruited children aged ≤5 years hospitalized for HAdV pneumonia from nine hospitals in China. Subjects were randomized 1:1 to receive either aerosol inhalation of IFN-α2b (200 000 IU/kg) plus routine supportive treatment or aerosolized normal saline plus supportive therapy, with a 7-day treatment period. Primary outcome indexes were viral DNA negative conversion rate and overall effective rate, while the secondary indexes included disappearance time of various symptoms, overall symptom improvement rate (OSIR), and levels of inflammatory indicators (TNF-α, IL-6, CRP, and LDH). Noteworthily, besides common clinical and laboratory indexes, two crucial effector proteins [2′,5′-oligoadenylate synthetase (OAS), *β*2 microglobulin (*β*2M)] of IFN-mediated antiviral responses were assayed using the ELISA method. Additionally, safety assessment parameters consisted of vital signs, biochemical indicators of liver and kidney function (ALT, AST, urea, creatinine), and incidence of adverse events.

**Results:**

A total of 140 eligible subjects were enrolled, including 70 in the IFN-α2b group and 70 in the control group. After 7-day therapy, compared to control group, the IFN-α2b group demonstrated significant superiority in therapeutic effects, including shorter disappearance time of various clinical symptoms, greater OSIR (0.84 ± 0.14 vs. 0.72 ± 0.11, *P* = 0.039), lower levels of inflammatory indicators (TNF-α, IL-6, CRP), and higher rates of viral DNA negative conversion [71.4 vs. 52.9%, RR (95% CI) = 1.35 (1.04–1.76), *P* = 0.023] and overall clinical effective [97.1 vs. 85.7%, RR (95% CI) = 1.13 (1.02–1.26), *P* = 0.016] with a more prominent clinical cure proportion [70.0 vs. 48.6%, RR (95% CI) = 1.44 (1.08–1.92), *P* = 0.010]. Meanwhile, higher serum OAS [85.1 (78.3–91.5) vs. 67.4 (63.5–71.0) U/mL, *P* = 0.003] and *β*2M [5.3 (4.8–6.5) vs. 3.8 (3.4–4.6) mg/L, *P* < 0.001] levels were confirmed in the IFN-α2b group than the control group after 7-day intervention. In terms of safety, there were no remarkable between-group differences regarding vital signs, liver/kidney function indexes, and incidence of adverse events.

**Conclusion:**

Aerosol inhalation of recombinant human IFN-α2b injection is safe and effective for treating pediatric HAdV pneumonia.

## Introduction

Human adenovirus (HAdV), a double-stranded non-enveloped DNA virus, is the common cause of respiratory infections among children worldwide, especially for those not exceeding 5 years of age^[1]^. According to the existing epidemiological data, HAdV infections account for 2.0–5.0% of the overall respiratory tract illness and 4.0–10.0% of pneumonia in children^[2,3]^. Although many cases with HAdV infections usually manifest as mild and self-limited symptoms of upper respiratory infections, about 28.7% of children infected by HAdV may develop pneumonia, with typical symptoms including cough, expectoration, wheezing, shortness of breath, and pulmonary rale^[4]^. Compared to other community-acquired pneumonia (CAP), HAdV-associated pneumonia progresses rapidly and is one of the significant contributors to child deaths. A recent large-scale study in the United States reported an overall mortality rate of 1.4–3.0% in pediatric HAdV-associated pneumonia^[5,6]^. For the severe cases who do not receive timely and effective treatments, the mortality may exceed 50.0% and even the survivors also are found to be closely associated with a high risk of long-term respiratory sequelae, such as bronchiolitis obliterans, bronchiectasis, and pulmonary interstitial fibrosis^[7,8]^. Due to the prominent disease burden and clinical severity, HAdV pneumonia has attracted increasing attention and concerns globally in recent years. However, no antiviral therapy is approved specifically for HAdV pneumonia. Despite the presence of ribavirin, cidofovir, acyclovir, and ganciclovir, they are not currently recommended in routine clinical practice because of their limited efficacy and uncertain safety^[9]^. Therefore, HAdV pneumonia in children remains a serious challenge to pediatricians, who urgently need to explore an efficient and reliable antiviral intervention strategy.

Interferons (IFNs), a family of multifunctional cytokines, play essential roles in host defense, particularly the IFN-α that is endowed with a potent antiviral activity^[10,11]^. IFN-α establishes an antiviral cellular state through the induction of several antiviral proteins, thus controlling the viral replication and spread. Previous studies have demonstrated that deficiency or delayed expression of type-I IFN is one of the reasons for recurrent viral respiratory infections and severe progression in young children^[12,13]^. Traditionally, IFN-α has been administered via intramuscular injection in patients; however, this route is often linked to some systemic side effects, such as fever, myalgia, and even the potential for further immunological injury, all of which greatly limit its clinical application, especially in the pediatric population^[14,15]^. As an alternative, aerosol inhalation of IFN-α is considered a theoretically superior route of administration. Although it may be linked with potential local risks (such as upper-airway irritation, mucosal dryness or discomfort, and reflex bronchospasm), this delivery method enables the drug to reach the respiratory tract directly, thereby optimizing local antiviral effectiveness while reducing systemic absorption and related side effects^[16]^. Nevertheless, although previous studies have demonstrated the significant therapeutic effects of IFN-α aerosol inhalation in various viral infections (including coronavirus, rhinovirus, respiratory syncytial virus, etc.)^[17,18]^, clinical data on the application of IFN-α aerosol inhalation in children with HAdV pneumonia still remain limited, with most available data derived from case reports. For example, Lin *et al*^[19]^ described a 10-month-old infant with severe HAdV pneumonia complicated by plastic bronchitis who improved after receiving aerosolized recombinant human IFN-α. Robust evidence from well-designed randomized controlled trials is still lacking, limiting its strength of recommendations for clinical practice.

**HIGHLIGHTS**

- Human adenovirus (HAdV) pneumonia represents one of the gravest threats to child health worldwide, but effectively and safely antiviral strategies for HAdV infections remain limited.
- First multicenter RCT to comprehensively evaluate the efficacy and safety of aerosol inhalation of recombinant human IFN-α2b injection in pediatric HAdV pneumonia.
- Aerosol inhalation of IFN-α2b significantly improved the clinical outcomes of pediatric HAdV pneumonia, with a low rate of adverse events.
- Two crucial effector proteins (OAS and *β*2M) of IFN-mediated antiviral responses were also examined, thus confirming and explaining, at the molecular level, the superior efficacy of aerosolized IFN-α2b for HAdV pneumonia.

Therefore, we performed this multicenter randomized controlled trial to evaluate the efficacy and safety of aerosol inhalation of recombinant human IFN-α2b injection in pediatric HAdV pneumonia, aiming to provide a scientific basis for optimizing the clinical management of such patients. Noteworthy, in addition to routine clinical and laboratory parameters, we also collected peripheral blood samples from patients before and after interventions to measure serum levels of two key antiviral proteins– 2′,5′-oligoadenylate synthetase (OAS) and *β*2 microglobulin (*β*2M) – which are critical effector molecules of the IFN-mediated antiviral responses. These biomarkers were assessed to provide further evidence and mechanistic insight for analyzing the clinical efficacy of aerosol inhalation of recombinant human IFN-α2b in the subject population. This work was compliant with the TITAN Guidelines 2025 for transparent artificial intelligence use^[20]^.

## Methods

### Study population and design

This was a prospective, multicenter, randomized, controlled trial, which was conducted in nine public tertiary hospitals in China, between 2023 and 2024. This study has been registered on the Chinese Clinical Trial Registry and was approved by the Ethics Committees of all participating hospitals. Written informed consent was received from the guardians of all participants, ensuring that all of them were informed of the main objective, protocol, benefits, and the potential risks of the present trial. This work has been reported in line with Consolidated Standards of Reporting Trials (CONSORT) Guidelines^[21]^.

The inclusion criteria of this study were as follows: (1) Children (aged ≤5 years) hospitalized for HAdV pneumonia with positive HAdV RT-PCR (nasopharyngeal swab specimens) reports, and the diagnosis was performed according to the *Guideline for Diagnosis and Treatment of Adenovirus Pneumonia in Children (2019 version)*^[9]^; (2) the interval from symptom onset to admission was within 48 h; and (3) patients who did not receive any other antiviral drugs or immunomodulators within 1 week prior to admission.

Besides, patients with one of the following conditions were excluded from this study: (1) patients with known allergy or other contraindication to IFNs; (2) patients with severe underlying conditions, including autoimmune disorders, inborn errors of immunity (IEI), congenital cardiac diseases, plastic bronchitis, bronchopulmonary dysplasia, liver/renal dysfunction, malignant tumors, severe malnutrition; (3) patients co-infected by other pathogens before enrollment. Pathogen testing was performed using a nasopharyngeal swab multiplex PCR Kit (Huanan Biological, Guangzhou, China), which covers 22 common respiratory pathogens, such as HAdV, influenza A virus, parainfluenza virus, respiratory syncytial virus, *Mycoplasma pneumoniae, Legionella pneumophila, Streptococcus pneumoniae, Klebsiella pneumoniae, Staphylococcus aureus*, etc.; (4) patients without the capability to receive aerosol inhalation; and (5) patients determined by physicians as unsuitable for the involvement of this trial.

### Randomization and allocation concealment

Before intervention, all eligible subjects were randomly assigned into the IFN-α2b group and the control group at a 1:1 ratio, according to the random numbers (simple randomization) generated by *SPSS* software version 22.0 (IBM Inc., Armonk, New York, USA). Study medications were assigned the same numbers corresponding to the randomization sequence. The numbered medications were dispensed exclusively by a nurse independent of this trial, ensuring that both subjects and investigators (treating physicians, outcome assessors, and researchers for data analysis) were blinded to the intervention options during the entire study period.

### Intervention

Patients in the IFN-α2b group were given routine symptomatic supportive treatment (such as antipyretic, rehydration, anti-inflammatory, antiasthmatic, oxygen therapies) and aerosol inhalation of recombinant human IFN-α2b injection (drug specific activity: 200 000 IU/μg) (Anke Biotechnology Co., Ltd, Anhui, China; Lot#: S20000013), which was administered at a dose of 200 000 IU/kg (diluted in 2.5 mL normal saline) twice daily, with a treatment duration of 7 consecutive days^[22]^.

Patients in the control group received the same routine therapy plus aerosol inhalation of 2.5 mL normal saline (twice daily for 7 days). Standardized compressor nebulizers (Pari GmbH, Starnberg, Germany) with a produced droplet size of 3.2 µm were employed uniformly across all participating centers to deliver treatment utilizing an identical nebulization process.

### Efficacy-safety evaluation

Blood samples were obtained from all subjects before and after the 7-day treatment, and were used for routine hematological tests, biochemical tests, and examination of two antiviral biomarkers (OAS, *β*2M). Of which, routine laboratory indicators were examined using automated hematology/biochemical analyzers in clinical laboratory departments, while serum OAS and *β*2M levels were assayed using 2,5-OAS (GTX, Shanghai, China; Lot#: YS02198B) and *β*2M (Abcam, Cambridge, UK; Lot#: AB99977) ELISA KITs according to the manufacturers’ instructions, respectively. Vital signs [body temperature (BT), respiratory rate (RR), heart rate (HR), diastolic blood pressure (DBP)] and clinical symptoms (cough, expectoration, wheezing, shortness of breath, pulmonary rale) were recorded daily throughout the 7-day study period. Additionally, nasopharyngeal swab specimens were collected after treatment to determine (RT-PCR approach) whether viral DNA was still present. These above data were collected and analyzed for efficacy-safety assessments of IFN-α2b aerosol inhalation.

#### Primary and secondary outcome indexes for efficacy

The primary outcome measures after 7-day treatment included the overall effective rate and viral negative conversion rate. Specifically, (1) clinical efficacy was divided into “clinical cure,” “clinical control,” and “ineffective,” respectively. Among which, “clinical cure” was defined as the disappearance of relevant clinical symptoms and signs, “clinical control” was defined as the improvement in relevant symptoms and signs, while patients with unimproved or even aggravated clinical abnormalities after treatment were classified to “ineffective.” The overall effective rate = clinical cure rate + clinical control rate. (2) Viral DNA negative conversion was defined by the negative results of nasopharyngeal RT-PCR test for HAdV after 7 days of treatment. Viral negative conversion rate = number of negative cases/total number of cases × 100%.

The secondary efficacy measures consisted of disappearance time of various symptoms (cough, expectoration, wheezing, shortness of breath, pulmonary rale), daily score of single symptom (a four-grade scoring scale: 0 point for absence of symptoms; 1 for mild; 2 for moderate; and 3 for severe) (Supplemental Digital Content Table S1, available at: http://links.lww.com/JS9/G598)^[17,23]^, overall symptom improvement rate [OSIR, OSIR = (pretreatment score of total symptoms − posttreatment score of total symptoms)/pretreatment score of total symptoms], changes in levels of inflammatory indicators [tumor necrosis factor-α (TNF-α), interleukin-6 (IL-6), C-reactive protein (CRP), lactate dehydrogenase (LDH)] and antiviral biomarkers (OAS, *β*2M).

#### Safety assessment

The safety indexes were comprised of vital signs (BT, RR, HR, DBP), critical biochemical parameters [alanine aminotransferase (ALT), aspartate aminotransferase (AST), urea, creatinine], and incidence of adverse events [rash, sore throat, gastrointestinal symptoms, leukopenia (i.e., leucocyte count < 4 × 10^9^ L)]. Of which, the severity of adverse events was classified as mild, moderate, or severe, based on the *Common Terminology Criteria for Adverse Events v4.03*^[24]^.

### Statistical analysis

Statistical analysis and data visualization were conducted using *R* software version 3.5.1 (R Foundation for Statistical Computing, Vienna, Austria) and *GraphPad Prism* version 7 (GraphPad Software Inc., San Diego, California, USA). Categorical data were expressed as frequencies (*n*) with percentages (%) and compared utilizing Pearson’s chi-square or Fisher’s exact test. Continuous data with normal distribution were presented as the means ± standard deviation (SD) and compared using an independent sample *t*-test, while non-normally distributed data were shown as medians with interquartile range (IQR) and compared applying Mann–Whitney *U* test. For continuous data measured repeatedly, including daily symptom scores and vital signs (BT, RR, HR, DBP), were analyzed by two-way repeated measures analysis of variance (rmANOVA), which included group-time interaction analysis. A two-sided *P*-value less than 0.05 was considered to be statistically significant.

## Results

### Baseline characteristics

**Figure 1.**
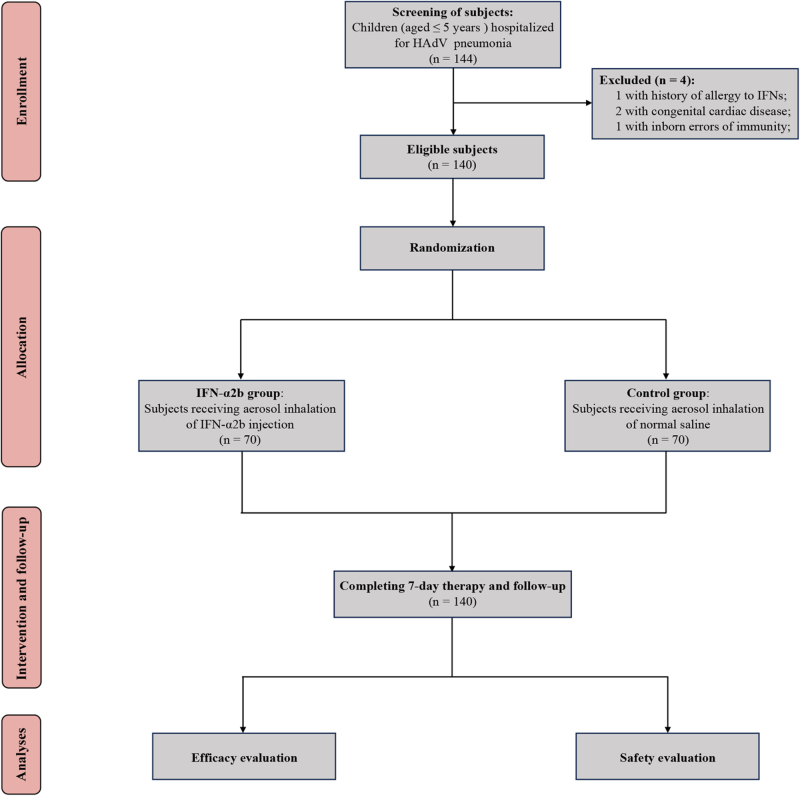
Flowchart of the trial. HAdV, human adenovirus; IFN-α2b, interferon-α2b.

A total of 140 eligible subjects were finally enrolled in this study (Fig. 1). Among which, 70 cases were included into the IFN-α2b group with a median age of 3.3 (2.1, 4.4) years and a male proportion of 62.9% (44/70), the remaining 70 cases were assigned into the control group having a median age of 2.8 (2.2, 4.2) years and a male proportion of 55.7% (39/70). Details regarding the baseline characteristics were summarized in Table 1, which showed no significant differences in demographic characteristics, clinical scores, vital signs, and laboratory data between the two groups before the treatment (all *P* > 0.05).

**Table 1:** Baseline characteristics of all subjects before treatment.

Characteristics	IFN-α2b group (*n* = 70)	Control group (*n* = 70)	*P-v*alue
Age, months, median (IQR)	3.3 (2.1, 4.4)	2.8 (2.2, 4.2)	0.468
Male, *n* (%)	44 (62.9)	39 (55.7)	0.390
BMI, kg/m^2^, median (IQR)	18.7 (16.6, 19.8)	19.1 (16.3, 22.1)	0.527
History of preterm birth, *n* (%)	9 (12.9)	7 (10.0)	0.595
Duration of symptoms prior to admission, h, median (IQR)	16.0 (10.0, 24.0)	18.0 (10.0, 27.0)	0.218
Daily scores of single symptom, mean ± SD			
Cough	2.27 ± 0.31	2.20 ± 0.25	0.874
Expectoration	2.29 ± 0.27	2.21 ± 0.34	0.796
Wheezing	1.89 ± 0.42	1.96 ± 0.77	0.505
Shortness of breath	1.91 ± 0.31	1.83 ± 0.29	0.537
Pulmonary rale	1.96 ± 0.38	1.90 ± 0.32	0.780
Pretreatment score of total symptoms, mean ± SD	8.94 ± 1.79	8.50 ± 1.83	0.845
Vital signs, mean ± SD			
BT, °C	36.77 ± 0.68	36.62 ± 0.57	0.914
RR, breaths/min	28.83 ± 3.75	28.90 ± 4.10	0.382
HR, beats/min	110.76 ± 7.42	111.32 ± 6.04	0.516
DBP, mmHg	56.34 ± 4.17	55.95 ± 7.69	0.704
Laboratory parameters, median (IQR)			
TNF-α, pg/mL	19.3 (15.5, 25.0)	20.5 (15.6, 28.3)	0.723
IL-6, pg/mL	19.5 (13.4, 30.3)	19.1 (10.9, 29.4)	0.129
CRP, mg/L	13.2 (10.6, 18.3)	12.7 (11.7, 16.5)	0.758
LDH, U/L	365.6 (311.5, 442.0)	348.8 (304.1, 418.9)	0.560
OAS, U/mL	50.4 (48.9, 54.8)	48.9 (44.3, 53.2)	0.480
*β*2M, mg/L	2.1 (2.0, 2.4)	2.2 (2.0, 2.6)	0.773
ALT, U/L	28.0 (24.0, 36.0)	27.0 (24.0, 34.0)	0.550
AST, U/L	24.0 (21.0, 28.0)	25.0 (21.0, 30.0)	0.639
Urea, mmol/L	4.2 (3.5, 4.9)	4.1 (3.2, 4.7)	0.672
Creatinine, µmol/L	33.6 (29.3, 39.3)	34.7 (31.8, 41.0)	0.830

ALT, alanine aminotransferase; AST, aspartate aminotransferase; BMI, body mass index; BT, body temperature; CRP, c-reactive protein; DBP, diastolic blood pressure; HR, heart rate; IL-6, interleukin-6; IQR, interquartile ranges; LDH, lactate dehydrogenase; OAS, 2′,5′-oligoadenylate synthetase; RR, respiratory rate; SD, standard deviation; TNF-α, tumor necrosis factor-α; *β*2M, *β*2 microglobulin.

### Primary outcome indexes for efficacy

As shown in Table 2, compared to the control group, the IFN-α2b group displayed higher rates of clinical cure cases [70.0% (49/70) vs. 48.6% (34/70), risk ratio (RR) = 1.44, 95% CI = 1.08–1.92, number needed to treat (NNT) = 5, *P* = 0.010] and overall effective cases [97.1% (68/70) vs. 85.7% (60/70), RR = 1.13, 95% *CI* = 1.02–1.26, NNT = 9, *P* = 0.016] after therapy. Furthermore, 50 cases in the IFN-α2b group and 37 cases in the control group were determined to be HAdV DNA negative after treatment, suggesting a greater viral negative conversion rate in the IFN-α2b group than the control group [71.4% (50/70) vs. 52.9% (37/70), RR = 1.35, 95% CI = 1.04–1.76, NNT = 6, *P* = 0.023].

**Table 2:** Clinical outcome and viral negative conversation after treatment.

Characteristics	IFN-α2b group (*n* = 70)	Control group (*n* = 70)	RR, 95% CI	NNT	*P-*value
Overall effective, *n* (%)	68 (97.1)	60 (85.7)	1.13 (1.02–1.26)	9	0.016
Clinical cure, *n* (%)	49 (70.0)	34 (48.6)	1.44 (1.08–1.92)	5	0.010
Clinical control, *n* (%)	19 (27.1)	26 (37.1)	0.73 (0.45–1.19)	–	0.205
Ineffective, *n* (%)	2 (2.9)	10 (14.3)	0.20 (0.05–0.88)	–	0.016
HAdV DNA negative conversion	50 (71.4)	37 (52.9)	1.35 (1.04–1.76)	6	0.023

DNA, deoxyribonucleic acid; HAdV, human adenovirus; NNT, number needed to treat; RR, risk ratio.

### Secondary outcome indexes for efficacy

**Figure 2.**
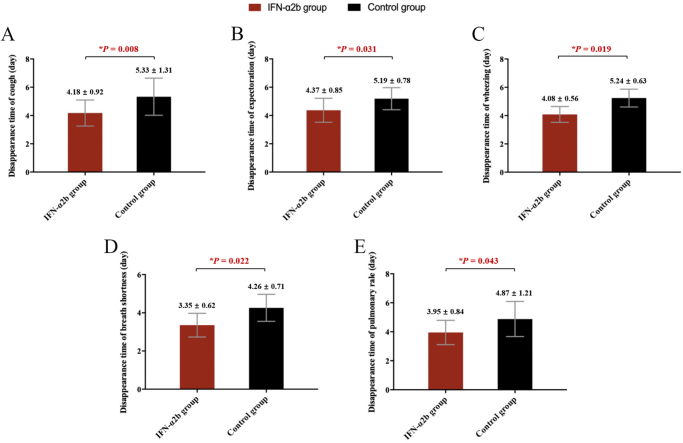
Disappearance time of clinical symptoms, consisting of (A) cough; (B) expectoration; (C) wheezing; (D) shortness of breath; and (E) pulmonary rale. Significantly statistical difference was labeled in red *P.

As presented in Figure 2, the disappearance time of all clinical symptoms, including cough, expectoration, wheezing, shortness of breath, and pulmonary rale, in the IFN-α2b group was significantly shorter than that in the control group (all *P* < 0.05).

**Figure 3.**
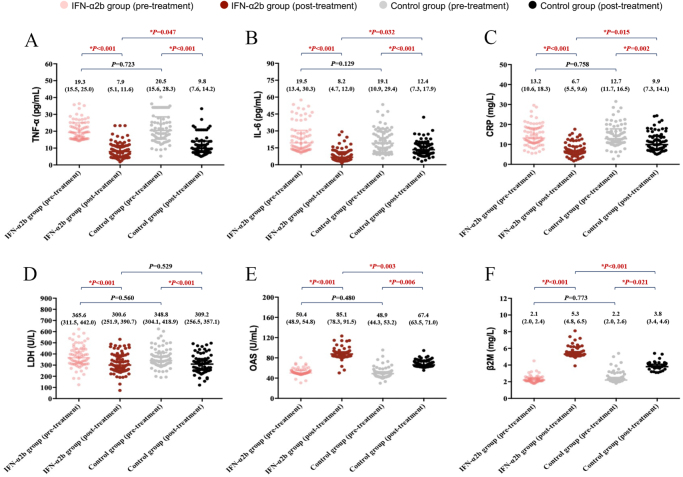
Laboratory parameters of patients in the two groups before and after 7-day treatment. (A) Levels of tumor necrosis factor-α (TNF-α), (B) interleukin-6 (IL-6), (C) C-reactive protein (CRP), (D) lactate dehydrogenase (LDH), (E) 2′,5′-oligoadenylate synthetase (OAS), and (F) *β*2 microglobulin (*β*2M). The red *P denoted significantly statistical difference.

In terms of laboratory parameters (Fig. 3), compared to their baseline data, both IFN-α2b and control groups showed significantly decreased levels of four inflammatory indicators (TNF-α, IL-6, CRP, and LDH) and elevated expression of two antiviral biomarkers (OAS and *β*2M) after 7-day treatment. More importantly, marked post-treatment differences in the above parameters, except for LDH (*P* = 0.529), were observed between the two groups: the IFN-α2b group exhibited lower post-treatment levels of TNF-α [7.9 (5.1, 11.6) vs. 9.8 (7.6, 14.2) pg/mL, *P* = 0.047], IL-6 [8.2 (4.7, 12.0) vs. 12.4 (7.3, 17.9) pg/mL, *P* = 0.032], and CRP [6.7 (5.5, 9.6) vs. 9.9 (7.3, 14.1) mg/L, *P* = 0.015], alongside higher expression of OAS [85.1 (78.3, 91.5) vs. 67.4 (63.5, 71.0) U/mL, *P* = 0.003] and *β*2M [5.3 (4.8, 6.5) vs. 3.8 (3.4, 4.6) mg/L, *P* < 0.001], than those in the control group.

**Figure 4.**
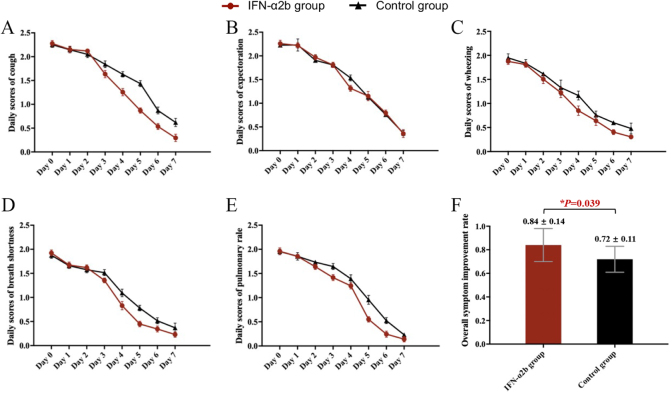
Daily scores of single clinical symptom during this trial and overall symptom improvement rate (OSIR) after treatment in the two groups. (A) Daily scores of cough, (B) daily scores of expectoration, (C) daily scores of wheezing, (D) daily scores of breath shortness, (E) daily scores of pulmonary rale, and (F) comparison of OSIR of the two groups after treatment. *Day 0* indicated “before treatment” and *Day 7* denoted “after completion of treatment.”

Moreover, all daily scores of clinical symptoms in both IFN-α2b and control groups presented gradual downward trends during the study period, achieving significant lower scores after treatment (scores in Day 7) than those before treatment (scores in Day 0) (all *P* < 0.001) (Fig. 4). Noteworthily, there were statistical discrepancies in the changes of daily symptom scores between the two groups: except for expectoration (*P* > 0.05), the IFN-α2b group showed significantly superior efficacy to the control group in reducing daily scores of cough, wheezing, shortness of breath, and pulmonary rale (all *P* < 0.05). As for the analysis of OSIR after treatment, the IFN-α2b group achieved an OSIR of 0.84 ± 0.14, which was greater than the 0.72 ± 0.11 in control group (*P* = 0.039).

### Safety assessment

**Figure 5.**
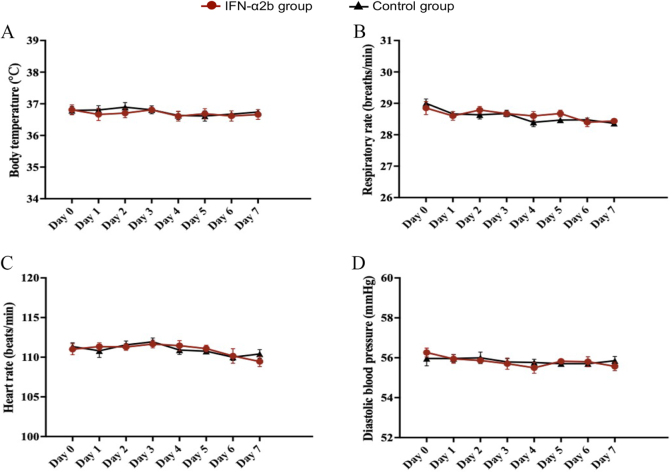
Vital signs of participants in IFN-α2b and control groups during this trial, including (A) body temperature (BT), (B) respiratory rate (RR), (C) heart rate (HR), and (D) diastolic blood pressure (DBP).

Vital signs were monitored and recorded daily. BT, RR, HR, and DBP of subjects in the two groups basically undulated within the physiological variability during the trial, and also did not show pronounced differences between the IFN-α2b and control groups (all *P* > 0.05) (Fig. 5).

**Figure 6.**
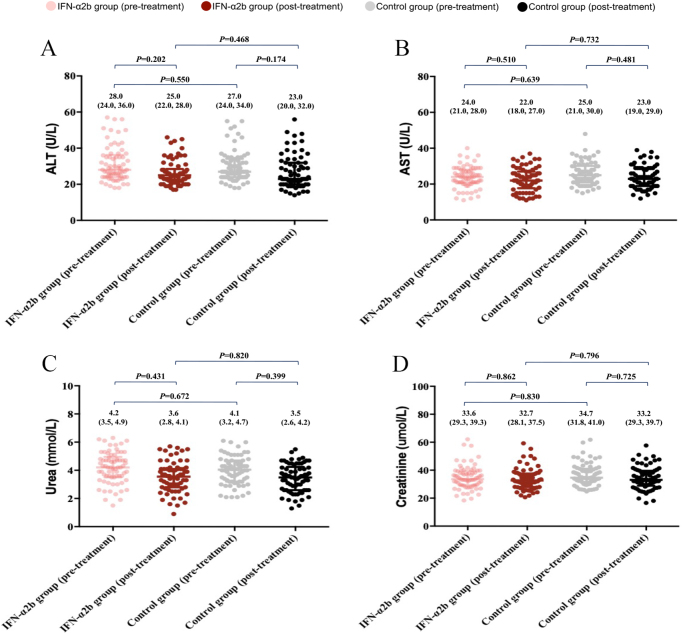
Biochemical indicators of patients in IFN-α2b and control groups before and after 7-day treatment, consisting of (A) alanine aminotransferase (ALT); (B) aspartate aminotransferase (AST); (C) urea; and (D) creatinine. All differences observed here were non-significant, all of which were indicated by black *P.*

In addition, slight declines in the levels of ALT, AST, urea, and creatinine were noted in the both two groups after 7-day treatment compared with before treatment, but these were not statistically significant (Fig. 6). Meanwhile, there were also no prominent between-group differences regarding the above four biochemical indicators in the IFN-α2b and control groups, neither pretreatment nor posttreatment (Fig. 6).

Analyses of adverse events were summarized in Table 3. Specifically, the IFN-α2b group showed five patients developing adverse events, including one with rash, one with sore throat, two with gastrointestinal symptoms, and one with leukopenia (a leukocyte count of 3.8 × 10^9^/L, without any associated clinical symptoms), indicating an adverse event incidence of 7.1% (5/70). Among these, only the occurrence of mild sore throat in a patient was likely related to IFN-α2b aerosol inhalation, possibly due to local airway mucosa irritation caused by the drug. The symptom resolved spontaneously after 1 day of rest. For the control group, there was an adverse event incidence of 4.3% (3/70), including one case developing a sore throat and two having gastrointestinal symptoms. No significant discrepancy regarding incidences of adverse events existed between groups (*P* = 0.466). Besides, all observed adverse events were mild, resolved without additional intervention, and did not lead to withdrawal from this trial.

**Table 3:** Adverse events during the trial.

	Rash	Sore throat	Gastrointestinal symptoms	Leukopenia	Total
IFN-α2b group (*n* = 70)					
Number of cases	1 (1.4)	1 (1.4)	2 (2.9)	1 (1.4)	5 (7.1)
Severity	Mild	Mild	Mild	Mild	–
Occurrence time (day)	4th	2nd	3rd and 7th, respectively	3rd	–
Relation to treatment	Unrelated	Possible	Unrelated	Unrelated	–
Control group (*n* = 70)					
Number of cases	0	1 (1.4)	2 (2.9)	0	3 (4.3)
Severity	–	Mild	Mild	–	–
Occurrence time (day)	–	6th	2nd and 3rd, respectively	–	–
Relation to treatment	–	Unrelated	Unrelated	–	–
*P-*value					
–	–	–	–	–	0.466

–, Not applicable.

## Discussion

HAdV pneumonia has long been a major health threat to children worldwide, and optimizing the treatment strategy is one of the keys for solving this issue. Under this context, we performed this multicenter study to prospectively assess the efficacy and safety of recombinant human IFN-α2b aerosol inhalation in pediatric HAdV pneumonia, with the objective of providing a reliable reference regimen for the clinical antiviral treatment of such patients. Final results of the present study indicated that aerosolized IFN-α2b significantly improved the clinical outcomes of children hospitalized for HAdV pneumonia, with a low rate of adverse events. Noteworthily, besides common clinical and laboratory evaluation, two crucial effector proteins (OAS and *β*2M) of IFN-mediated antiviral responses were also examined in this trial. The result of examination revealed higher serum levels of OAS and *β*2M in the IFN-α2b group than those in the control group after completing a 7-day intervention, which also confirmed and explained, at the molecular level, the superior clinical effects observed in the IFN-α2b group. All participants were enrolled within 48 h of symptom onset. This early enrollment ensured that all subjects were in a comparable phase of illness, minimizing variability in disease progression at baseline. Consequently, the observed antiviral effects and clinical outcomes can be more reliably attributed to the intervention rather than differences in disease stage. To our knowledge, this is the first multicenter randomized controlled trial that comprehensively evaluated the efficacy and safety of aerosol inhalation of recombinant human IFN-α2b injection in children with HAdV pneumonia.

As shown in previous research^[17]^, aerosol inhalation of IFN-α2b has achieved remarkable effects in the treatment of several viral pneumonias, such as pneumonia caused by the common respiratory syncytial virus or rhinovirus. Encouragingly, as an important supplement to this field, our study revealed a satisfactory efficacy of IFN-α2b aerosol inhalation on HAdV pneumonia. Specifically, compared to the control group, the IFN-α2b group displayed notable superiority in terms of disappearance time of typically clinical symptoms (cough, expectoration, wheezing, shortness of breath, pulmonary rale), OSIR, and viral negative conversion rate. Of which, OSIR was calculated as the percentage reduction in total symptom scores from baseline to posttreatment, defined as OSIR = pretreatment total symptom score − posttreatment total symptom score)/pretreatment total symptom score. Higher OSIR value in the IFN-α2b group indicated greater symptom improvement, reflecting the superior therapeutic efficacy. Furthermore, after completing the therapy, the proportions of patients defined as “overall effective” (97.1%) and “clinical cure” (70.0%) in the IFN-α2b group were significantly higher than those in the control group (85.7 and 48.6%, respectively). The accelerated improvements in clinical outcomes in the IFN-α2b group align with the known antiviral property of IFN-α2b, which mainly acts through inducing and upregulating the expression of antiviral effectors collectively termed interferon-stimulated genes, such as OAS and *β*2M^[10]^. And, this molecular mechanism also explains the prominent higher levels of OAS and *β*2M in the IFN-α2b group than those detected in the control group after the treatment.

IFNs have been acknowledged as the critical components of the innate immune response, providing the first line of defense against viral infection. Among the IFN-α-mediated antiviral-signaling transduction, OAS is undoubtedly one of the most crucial enzymes^[25]^. Viral double-stranded RNA (dsRNA) could activate IFN-α-induced OAS, which produces 2′-5′-linked oligoadenylates (2–5As) to specifically stimulate the latent RNaseL, thereby inhibiting the synthesis of viral-associated proteins and the replication of virus^[26,27]^. The supplementation of exogenous IFN-α2b thereby enhances antiviral capability of the body by increasing the production of OAS.

In addition, another antiviral biomarker selected in our study is the *β*2M, which is a key subunit of the major histocompatibility complex class I (MHC-I) and plays an important role in the maintenance of structural stability, thus allowing the viral peptides to be accurately and stably presented to cytotoxic T lymphocytes (CTLs)^[28]^. However, HAdV has developed multiple immune evasion mechanisms during infection, such as manipulating MHC-I expression or producing viral proteins that directly inhibit antigen processing and presentation^[29,30]^, leading to declined recognition and destruction of HAdV-infected cells by CTLs. As a critical modulation, IFN-α can upregulate the expression of *β*2M and MHC-I, thereby helping the immune system target and eliminate the infected cells by reinforcing presentation of viral antigens to CTLs^[31]^. Hence, *β*2M could serve as an indirect indicator of antiviral efficacy induced by IFN-α, where the elevation of *β*2M corresponds to the heightened immune responses against viral infection. In conclusion, during the course of treatment, higher expression levels of the two downstream direct/indirect indicators (OAS and *β*2M) of IFN-α pathway activation were closely associated with more favorable clinical outcomes. Of course, there are several other IFN pathway-related marker proteins, such as myxovirus resistance protein A (MxA). However, previous studies suggested that MxA may be more suitable for RNA virus infections, like respiratory syncytial virus, influenza virus, and hepatitis C virus^[32–34]^. Besides, its short detection window period further improves the detection difficulty and limits the reliability of results. Therefore, we ultimately selected OAS and *β*2M as the IFN-associated antiviral biomarkers for this study. However, it should be noted that, although the observed trends in biomarker changes provided biologically meaningful insights into the antiviral effects of the drug, these findings are exploratory in nature and warrant further mechanistic studies to confirm the associated conclusions.

In this trial, TNF-α, IL-6, CRP, and LDH were monitored as secondary outcome indexes for treatment efficacy, and these four indicators are widely recognized to be commonly elevated in HAdV infections and associated with disease severity^[35]^. In our findings, except for LDH, the other three indicators showed significantly lower posttreatment levels in the IFN-α2b group than the control group, suggesting that IFN-α2b aerosol inhalation may successfully control the inflammatory responses, which was consistent with the better clinical improvements observe in patients of IFN-α2b groups. The reasonability of this consequence and inference is also further supported by the known anti-inflammatory role of IFN-α that could control the local infiltration of immune cells and intensity of the inflammatory responses via inhibiting the excessive release of inflammatory mediators^[36]^. Thus, integrating laboratory findings with clinical outcomes highlights that aerosolized IFN-α2b modulates pro-inflammatory biomarkers, effectively contributing to relief of respiratory inflammation and promoting recovery, particularly given that the aerosol inhalation approach can increase the local concentration of IFN-α2b.

Safety assessment has always been a crucial topic in clinical trials of drugs, especially for pediatric patients. Excitingly, the safety of aerosolized IFN-α2b in this trial was generally favorable. In previous clinical practice, IFN-α2b was mostly administered by intramuscular injection, which may present several systemic side effects, particularly fever^[37]^. Throughout our study, the vital signs, including body temperature, remained overall stable in subjects who received IFN-α2b aerosol inhalation, without significant differences compared to the controls. This improvement might largely be attributed to the reduced systemic absorption of IFN-α2b under aerosol inhalation compared to the injection route. In terms of critical biochemical parameters (ALT, AST, urea, and creatinine), there were no marked post-treatment abnormalities and between-group differences, suggesting no prominent indications of hepatic or renal toxicity. Furthermore, no severe adverse events were reported during this study; all observed events were mild, transient, and did not require discontinuation of the treatment. The total incidences of adverse events were comparable across the IFN-α2b and control groups. In conclusion, based on these findings, IFN-α2b aerosol inhalation was safe and well-tolerated among children with HAdV pneumonia.

Despite the above promising findings, this trial had several limitations. First, although our study possessed a multicenter design, the sample size was relatively small, which may limit the ability to detect rare but potentially serious adverse events. Meanwhile, the short trial duration may preclude evaluation of long-term outcomes. Second, due to the lack of published reliable treatment data, *a priori* sample size calculation was not performed; the actual sample size was determined by practical feasibility. Third, while standard supportive care was set up, intercenter variability in its actual implementation may constitute a potential confounder. Fourth, despite the double-blind design, subjective symptom scoring may still be susceptible to observer bias. Fifth, findings of this trial were specific to a pediatric population aged ≤5 years with early disease presentation, which warrants caution in extrapolating the results to older children or those with different disease stages. Another limitation of our study was the absence of more meticulous, regular quantitative measurements of viral load during the study period; thus, we were unable to construct a dynamic curve of viral decay under IFN-α2b intervention. Quantifying IFN-α2b’s antiviral impact would further strengthen any causal inference regarding the antiviral efficacy of nebulized IFN-α2b in pediatric HAdV pneumonia. Therefore, building on the current results, larger-scale in-depth research incorporating scheduled HAdV load monitoring, broader subject populations, and a longer follow-up period should be conducted in the future to further confirm and extend our findings.

## Conclusions

In summary, this study preliminarily offers scientific evidence that aerosol inhalation of recombinant human IFN-α2b injection is an effective and safe antiviral intervention for children hospitalized for HAdV pneumonia. This localized administration strategy presents a promising and practical approach for delivering IFN-α2b efficiently and safely to the respiratory tract, constituting an essential component of optimizing clinical management for such patients. Furthermore, on the basis of the current findings, future studies with larger sample sizes, longer-term follow-up, refined age stratifications, and quantitative viral load monitoring should be conducted to comprehensively confirm the therapeutic efficacy and safety, and further elucidate treatment response dynamics in pediatric HAdV pneumonia.

## Data Availability

Not applicable.

## Research registration unique identifying number (UIN)

This study has been registered on the Chinese Clinical Trial Registry (http://www.chictr.org.cn/, registration number: ChiCTR2100054534).
